# The stimulating role of syringic acid, a plant secondary metabolite, in the microbial degradation of structurally-related herbicide, MCPA

**DOI:** 10.7717/peerj.6745

**Published:** 2019-04-10

**Authors:** Magdalena Urbaniak, Elżbieta Mierzejewska, Maciej Tankiewicz

**Affiliations:** 1 Faculty of Food and Biochemical Technology, Department of Biochemistry and Microbiology, University of Chemistry and Technology Prague, Prague, Czech Republic; 2 Faculty of Biology and Environmental Protection, Department of Applied Ecology, University of Lodz, Lodz, lodzkie, Polska; 3 Department of Environmental Toxicology, Faculty of Health Sciences, Medical University of Gdańsk, Gdańsk, Poland

**Keywords:** Phenoxy herbicide, PSM, *tfdA*, MCPA, Biodegradation, Syringic acid

## Abstract

The ability of microorganisms to degrade xenobiotics can be exploited to develop cost-effective and eco-friendly bioremediation technologies. Microorganisms can degrade almost all organic pollutants, but this process might be very slow in some cases. A promising way to enhance removal of recalcitrant xenobiotics from the environment lies in the interactions between plant exudates such as plant secondary metabolites (PSMs) and microorganisms. Although there is a considerable body of evidence that PSMs can alter the microbial community composition and stimulate the microbial degradation of xenobiotics, their mechanisms of action remain poorly understood. With this in mind, our aim was to demonstrate that similarity between the chemical structures of PSMs and xenobiotics results in higher micropollutant degradation rates, and the occurrence of corresponding bacterial degradative genes. To verify this, the present study analyses the influence of syringic acid, a plant secondary metabolite, on the bacterial degradation of an herbicide, 4-chloro-2-methylphenoxyacetic acid (MCPA). In particular, the presence of appropriate MCPA degradative genes, MCPA removal efficiency and changes in samples phytotoxicity have been analyzed. Significant MCPA depletion was achieved in samples enriched with syringic acid. The results confirmed not only greater MCPA removal from the samples upon spiking with syringic acid, and thus decreased phytotoxicity, but also the presence of a greater number of genes responsible for MCPA biodegradation. 16S rRNA gene sequence analysis revealed ubiquitous enrichment of the β-proteobacteria *Rhodoferax, Achromobacter, Burkholderia* and *Cupriavidus*. The obtained results provide further confirmation that plant metabolites released into the rhizosphere can stimulate biodegradation of xenobiotics, including MCPA.

## Introduction

The introduction of newly-synthesized hazardous organic compounds (xenobiotics) in agriculture, such as pesticides and the herbicides used as weed control agents has been associated with a deterioration of ecosystem quality. This has aroused a great deal of research interest, not only regarding the potential for toxic effects on the environment and the occurring organisms, but also regarding methods to safely eliminate these xenobiotics from the environment (Smith et al., 1994).

Phenoxy acid herbicides, such as MCPA, have been widely used for agricultural purposes since their development in the 1940s. In Europe, MCPA has been among the five top-selling herbicides for the last five decades (Eurostat, 2016). It is most commonly used to protect wheat, one of the most widely-cultivated crops in the EU (Eurostat, 2016). The relatively large amounts applied (1,500–2,000 g/ha), together with its unrestricted use during cold autumns when the degradation rate is slow, have resulted in frequent contamination of soil and water, providing a potential risk to wildlife and aquatic ecosystems (López-Piñeiro et al., 2013). Moreover, MCPA demonstrates greater persistence in the acidic soils (López-Piñeiro et al., 2013) which cover most of the EU-27 area. This exacerbates the adverse effect of MCPA on the environment and leads to increased inhibition of plant development, enhanced soil toxicity and surface- and groundwater contamination (Pereira, Cerejeira & Espírito-Santo, 2000; Mierzejewska, Baran & Urbaniak, 2018).

Hence, there is a need to identify nature-based methods that enable effective elimination of MCPA from the environment. One such approach is bioremediation: the process of using organisms to neutralize or remove contamination from given matrices.

A particularly promising approach to the biodegradation of MCPA in soil is by the use of indigenous microbiota harboring desirable catabolic genes. The first step in the phenoxy herbicide biodegradation pathway is initiated by α-ketoglutarate-dependent dioxygenase, which is encoded by *tfdA* or *tfdA*-like genes (Batogliu-Pazarbasi et al., 2013; Mcgowan et al., 1998): for example, the *tfdAα* detected in α-proteobacteria (Itoh et al., 2004), and *tfdA Class I, II* and *III* belonging to the β- and γ-proteobacteria (Zhou, Wu & Xiang, 2014). The oxidative degradation of phenoxy acids, mediated by bacteria harboring *tfdA*-like genes, reduces the herbicide content in soil and mitigates its toxic effects on the environment.

However, although the ability of microorganisms to degrade MCPA can be exploited to develop cost-effective and eco-friendly bioremediation technology, this process might be very slow in some cases, such as in acidic soil or at the low temperatures characteristic of a temperate climate. Fortunately, the removal of MCPA and other recalcitrant xenobiotics from the environment can be accelerated by stimulating their bacterial degradation with PSMs, used by bacterial communities as a carbon sources to support their growth and stimulate the degradation of a particular xenobiotic (Musilova et al., 2016; Fraraccio et al., 2017). Moreover, studies have found that supplementing a sample with a compound that very closely resembles the target compound can significantly increase the rate of degradation (Hu et al., 2014; Musilova et al., 2016). Usually, PSMs serve as cometabolites: they provide the energy for a microorganism to carry out co-metabolism, where a xenobiotic is being degraded as a secondary substrate. Common examples of cometabolites are biphenyl and polychlorinated biphenyls (PCBs) (Singer et al., 2000). In addition, PSMs can stimulate the induction of desirable genes involved in the catabolic pathway of a given xenobiotic (Uhlik et al., 2013; Musilova et al., 2016).

Hence, the aim of the present study was to demonstrate that similarity between the chemical structures of a PSM and a xenobiotic influences the degradation rates of the micropollutant. To verify this, the study analyzes the influence of syringic acid (one of the PSMs) addition on (1) the presence of herbicide-degrading genes (in this case, MCPA) and (2) MCPA depletion. Since MCPA is the most widely-used phenoxy herbicide applied to kill broadleaf weeds, and one with the greatest long-term effect on the environment, (3) changes in its phytotoxic properties during the experiment were assessed using two plant species: *Lepidium sativum* and *Sinapis alba*.

## Materials & Methods

### Herbicide

The herbicide used in the experiment was MCPA, C_9_H_9_ClO_3_ (≥95.0% purity, molecular weight 200.61 g/mol, 0.825 g/L water solubility at 20 °C, pKa value 3.07) obtained from Sigma-Aldrich.

### Plant Secondary Metabolite (PSM)

The PSM used in the study was syringic acid, C_9_H_10_O_5_ (≥95.0% purity, molecular weight 198.17 g/mol, 5.78 mg/mL water solubility at 25 °C, pKa value 4.34) obtained from Sigma-Aldrich.

### Mineral Salt Medium (MSM)

MSM was prepared according to Gaunt & Evans (1971). The medium consists of 1 g/L of KNO_3_, 0.5 g/L of K_2_HPO_4_ and MgSO_4_ 7 H_2_O, 0.05 g/L of NaCl and CaCl_2_, and 0.01 g/L of FeCl_3_. For MSM preparation, ultrapure water was used. In order to obtain sterile samples, the MSM was filtered through a microbiological Corning™ Disposable Vacuum Filter (0.22 µL).

### Soil characteristics

The soil was collected from a crop field in Wólka Wojsławska, Zduńska Wola district, Łódź voivodship, Central Poland (51°38′45.169″N, 18°56′14.582″E). The soil was obtained using a manual composite technique in which soil samples were taken from random sites within the area, and then, six to eight subsamples were mixed thoroughly in the laboratory.

The C, N and S concentrations were analyzed using an Elementar Vario MAX cube CNS analyzer. The total concentration of the macroelements Na, Mg, Ca, K, P, Na and of the heavy metals Fe, Mn, Ni, Cr, Zn, Pb, Cd were assessed using the ICP-OES method (atomic emission spectrometry in inductively coupled argon plasma) on an Optima 7300DV system (Perkin-Elmer). The bioavailable forms of phosphorus and potassium were assayed according to Egner-Reihm. The soil reaction was determined potentiometrically either in a suspension of water, or 1 M KCl solution. Note, however, that 1 M KCl simulates the soil solution better than water (Goulding, 2016). The general physicochemical parameters of the soil are presented in Table 1.

**Table 1:** Mean and standard deviation for physicochemical properties of the soil used for soil slurry preparation.

**Properties**	**Unit**	**Soil**
pH (H_2_O)		5.58
pH (KCl)		4.31
**Macroelements**		
N	%	0.10 ± 0.00
C	%	0.98 ± 0.02
S	%	0.02 ± 0.00
P	g/kg	0.62 ± 0.02
P_2_O_5_ (assimilable phosphorus)	mg/kg	151.5 ± 5.00
K	g/kg	1.01 ± 0.17
K_2_O (assimilable potassium)	mg/kg	34.5 ± 1.60
Na	g/kg	0.11 ± 0.00
Mg	g/kg	0.69 ± 0.02
Ca	g/kg	0.77 ± 0.06
**Heavy metals**		
Fe	g/kg	4.13 ± 0.10
Cr	mg/kg	10.18 ± 2.95
Mn	mg/kg	208.51 ± 9.92
Ni	mg/kg	4.07 ± 0.13
Cu	mg/kg	12.78 ± 0.37
Zn	mg/kg	41.00 ± 3.20
Cd	mg/kg	1.23 ± 0.11
Pb	mg/kg	13.53 ± 1.99

### Soil extract

The soil extract was prepared according to Strzelczyk (1968) with some modifications. Briefly, 500 g of soil was sieved through a 2 mm sieve and poured over with 1.5 L of water. The suspension was mixed and left at room temperature on a gentle shaker for 24 h. After that, the soil extract was decanted and filtered through Whatman paper. The extract used for sterile samples (controls) was further filtered through a Corning Disposable Vacuum Filter (0.22 µL).

### Microcosm setup

The study was conducted in bacterial cultures containing liquid Mineral Salt Medium (MSM) enriched with soil microorganisms derived from the agricultural soil extract (50%:50%, v/v), and amended with MCPA at concentrations of 0.1 and 0.5 mM, and syringic acid at a concentration of 0.25 mM.

Samples containing only sterile MSM, and sterile MSM + sterile soil extract were used as controls to assess the degree of physicochemical degradation.

The samples were incubated in darkness at 25 °C for 24 days.

Subsamples were collected four times during the incubation period, i.e., every six days, and examined for the presence of two bacterial 16S rRNA and the occurrence of five MCPA degradative genes (*tfdA*, *tfdAα* and *tfdA Class I, tfdA Class II* and *tfdA Class III*).

Changes in MCPA concentration and phytotoxicity were analyzed twice during the experiment, at the beginning and after the 24-day incubation period.

### Molecular analysis

#### Bacterial DNA extraction and genes amplification

Bacterial DNA extraction was performed using a EurX Bacterial & Yeast Genomic DNA Purification Kit, according to the manufacturer’s instructions. Polymerase Chain Reaction (PCR) was performed according to Poll et al. (2010), Bælum, Jacobsen & Holben (2010) and Bælum et al. (2006) with minor modifications regarding the annealing temperature (Table 2). The 20 µL reaction mixture contained sterile nuclease free H_2_O, 1 × PCR buffer, 3.5 mM MgCl_2_, 0.2 mM dNTP (Qiagen), 0.5 µM primers (Genomed), 0.1 mg/mL BSA (Qiagen), 1–2.5 U/µL Taq polymerase (Qiagen) and 15–30 ng of template DNA.

**Table 2:** Primer sequences used for amplification of the target 16S rRNA, *tfdA* and *tfdA*-like genes.

**Target gene**	**Primer (5′–3′)**	**Frag. size (bp)**	**Anneal. temp. (°C)**	**Ref.**
16S rRNA	341F: CCT ACG GGA GGC AGC AG	174	50	Poll et al. (2010)
	515R: ATT CCG CGG CTG GCA			
16S rRNA	^a^27F: GAGAGTTTGATCCTGGCTCA	1,300–1,400	52	Orphan et al. (2001)
	^a^1492R: GGTTACCTTGTTACGACTT			
*tfdA*	F: GAGCACTACGCRCTGAAYTCCCG	210	64	Bælum et al. (2006)
	R: CTTCGGCCACCGGAAGGCCT			
*tfdAα*	F: CSGAGTTCKSCGACATGCG	350	66	Bælum et al. (2006)
	R: GCGGTTGTCCCACATCAC			
*tfdA Class I*	F: GTGAGCGTCGTCGCAAAT	856	56	Bælum, Jacobsen & Holben (2010)
	R: GCATCGTCCAGGGTGGTC			
*tfdA Class II*	F: TGAGCATCAATTCCGAATACC	882	53	Bælum, Jacobsen & Holben (2010)
	R: AAGACTGACCCCGTGGACT			
*tfdA Class III*	F: TGAGCATCACTTCCGAATACC	856	56	Bælum, Jacobsen & Holben (2010)
	R: ACAGCGTCGTCCAACGTC			

**Notes.**

a Primers used for sequencing.

The bacterial DNA fragment was amplified using two primer pairs specific to bacterial 16S rRNA, and five sets of primers specific to the *tfdA* gene (Table 2) coding for an enzyme capable of degrading MCPA. The conditions for the PCR were as follows: 10 min at 95 °C for activating the enzyme; 26 cycles of 10 s at 94 °C for denaturation, 30 s at optimal annealing temperature (see Table 2), and one minute at 72 °C for elongation. A final 10 min step was performed at 72 °C for the final extension. PCR products were separated by 1.5% agarose gel electrophoresis in TBE buffer (40 mM TRIS-HCl, 3 mM sodium acetate, 1 mM EDTA, pH 7.9) using constant voltage (70 V), and visualized using ethidium bromide (2 mg/mL), with DNA M100-500 (MR75) and DNA M600-1000 (MR85) markers (DNA Gdańsk). The gels were documented using the Uvitec system (Cambridge, UK).

#### Nucleotide sequence analysis

The 16S rRNA gene fragments (1,300–1,400 bp) were amplified by PCR using thermostable Pfu DNA polymerase (ThermoScientific) and specific primers (see Table 2 for the primer sequences). The amplified 16S rRNA gene fragments were purified using a QIAGEX II Gel Extraction Kit (Qiagen) and subjected to sequencing. Homology searches were performed using the National Center for Biotechnology Information microbial and nucleotide BLAST network service (http://blast.ncbi.nlm.nih.gov/Blast.cgi) (Zhang et al., 2000) and Vector NTI AdvanceTM 9 software (Invitrogen).

### Analysis of MCPA concentration

The samples for determination of changes in MCPA concentration, associated with the ongoing microbiological and physicochemical degradation processes, were collected twice during the experiment: once at the beginning and once after a four-week incubation period. The MCPA concentration was analyzed using GC-MS TQ 8040 (Shimadzu, Japan) gas chromatograph equipped with split/splitless injector operating in a splitless mode at 250 °C and triple quadrupole mass spectrometer (MS) connected to “LabSolutions” software extended with Pesticide Smart Database (Shimadzu).

The injection volume of 2 µL was selected for all analyses. Helium (purity 99.999995%) was used as a carrier gas and was supplied by Air Products (Warsaw, Poland). The flow rate of carrier gas was 1 mL/min, with constant flow conditions being observed throughout. Chromatographic separation in standard conditions was performed, with temperature program from 70 °C to 240 °C (at 12 °C/min) with total duration of 14.17 min.

The mass spectrometer was operated in selected-ion-monitoring (SIM) mode with solvent delay of 5 min. MS conditions were the following: ion source temp. 200 °C, interface temperature 250 °C, ionization voltage 70 eV, emission current 150 µA. Three specific ions (141, 77 and 200) were selected for MCPA pesticide and were used to identify the compound. The first ion, yielding a more intensive signal, was used for measurement and the other two for confirmation.

In order to precisely determine MCPA residues in tested samples, prior to the validation study, mass spectrometer operating in the SCAN mode was firstly applied. This enabled to eliminate the false positives and additional errors.

The working standard solutions for the calibration study were prepared by spiking the tested samples with the standard solution in the concentration range of 0.01–100 µg/mL. The linear range for MCPA was studied by replicate analysis of the standard stock solutions. The linear regression value was calculated with the average peak areas of five replicate injections. The linear regression was in the range of 0.19–100 µg/mL with coefficient of determination of 0.9996. Coefficient of variability (percentage of relative standard deviation, CV %) was the average value of different concentrations in the linear range and was 1.1%, which is considered as good method precision.

The sensitivity of the method was considered in terms of limit of detection (LOD). LOD was calculated based on calibration functions and it was equal to 62.5 ng/mL. The limit of quantitation (LOQ) defined as 3 times the LOD was 0.19 µg/mL. Then, after determining the basic validation parameters, environmental samples were analyzed in 9 replications. For each sample, the mean value was calculated together with the relative standard deviation.

### Phytotoxicity assessment

The phytotoxicity of the soil samples was assessed by Phytotoxkit Test (Microbiotest Inc., Nazareth, Belgium), a commercial toxicity bioassay (Phytotoxkit, 2004). The test measures the degree of inhibition of root length of test species after three days of exposure to a soil sample in relation to a reference soil. For the purpose of this experiment, the dicotyledons *Lepidium sativum* (L.) and *Sinapis alba* were used as test plants. Uncontaminated, artificial Organisation for Economic Co-operation and Development (OECD) soil with no treatment (control) was used as a reference sample to assess the phytotoxicity of tested samples enriched with soil extract and/or MCPA and /or syringic acid. The samples were collected twice during the experiment: once at the beginning and once after a four-week incubation period to assess the changes in sample phytotoxicity associated with the ongoing microbiological and physicochemical degradation processes. The response of the test species was classified as toxic when the percentage effect of root growth inhibition was ≥20% (Persoone et al., 2003).

## Results and Discussion

### The influence of syringic acid on the presence of MCPA degradative genes

Microbial growth and their degradative activity are known to be stimulated by PSMs. Flavonoids, coumarins and terpenes can modify the chemical and physical properties of soil and serve as substrates or inducers of the pathways used for contaminant catabolism by soil microorganisms (Uhlik et al., 2013; Hu et al., 2014; Musilova et al., 2016). For example, in mulberry (*Morus* sp.), the PSMs (morusin, morusinol, and kuwanon C) have been found to support the growth of *Burkholderia* sp. LB400: a degrader of PCBs (Leigh et al., 2002). Similarly, the PSM l-carvone, excreted by spearmint (*M. spicata*) stimulated the co-metabolism of PCBs by *Arthrobacter* sp. strain B1B (Gilbert & Crowley, 1997). Similar results were obtained using carvone and salicylic acid (Singer et al., 2000). The presence of phenolic compounds, flavonoids and gibberellic acid promoted polycyclic aromatic hydrocarbon (PAH) biodegradation (Ely & Smets, 2017). Elevated biotransformation of cis-1,2-dichloroethylene was obtained under the presence of acetophenone, phenethyl alcohol, p-hydroxybenzoic acid and trans-cinnamic acid (Fraraccio et al., 2017).

In addition to acting as growth substrates for bacteria, it has been hypothesized that PSM may also stimulate the detoxification mechanisms taking place in some bacterial strains (Uhlik et al., 2013; Hu et al., 2014). The expression of degradative genes in bacteria is essential for the successful bioremediation of xenobiotics; however, little is known of the influence of PSMs on the induction of genes involved in catabolic pathways. Siciliano et al. (2002) reported greater induction of catabolic genes (*ndoB, alkB, xylE*) involved in the degradation of naphthalene in the rhizosphere soil of tall fescue (*F. arundinaceae*) than in unplanted soil. Master & Mohn (2001) reported salicylate to have an up-regulating effect on the expression of *bphA*, which encodes biphenyl dioxygenase in the PCB-degrader *Pseudomonas* sp. Cam-1. Hu et al. (2014) demonstrated the expression of *bphA* gene in *R. eutropha* H850 and *P. fluorescens* P2W amended with salicylic acid. In addition, it has been hypothesized that the structural similarity of selected xenobiotics and PSMs may have a profound impact on the biodegradation of given, structurally-related xenobiotics (Musilova et al., 2016; Hu et al., 2014).

In view of this, the present study investigates the role played by a PSM, syringic acid, in the degradation of a structurally-similar herbicide, MCPA, by microorganisms present in samples of agricultural soil. The selection of this particular xenobiotic and related PSM was based on the fact that MCPA is the most widely-used phenoxy herbicide in agricultural practice, and has often been studied as a model compound in environmental fate studies (Poll et al., 2010; Nielsen et al., 2011). Syringic acid, in turn, is a characteristic PSM of the *Cucurbitaceae* (Yoon, Chung & Thiruvengadam, 2015): plants known for their ability to take up a variety of organic xenobiotics from soil and translocate them to leaves and fruits (Greenwood, Rutter & Zeeb, 2011; Urbaniak et al., 2016). The structural similarity of syringic acid to MCPA indicates that it may enhance the degradation of the latter by indigenous soil microorganisms.

In our case, a soil extract was used to introduce the soil microbiota to the samples. The 16S rRNA genes fragments (174 bp; 1,300–1,400 bp), specific for bacteria, were found to be present in all samples and all variants, indicating the presence of an abundance of bacterial communities in the studied samples (Table 3). Then, to assess the impact of syringic acid on bacterial degradation of MCPA, the process determined by the functional genes from the *tfdA* cluster, the samples were spiked with herbicide (MCPA) and/or PSM (syringic acid). The results showed that of the studied MCPA degradative genes (*tfdA*, *tfdAα*, *tfdA Class I*, *Class II* and *Class III*) only *tfdA* was not detected in any sample; *tfdAα* and *tfdA Class II* and *III* were observed in 44% of samples (Table 3). The *tfdA Class III* gene was the most common, being detected in 38% of samples amended with soil extract without PSM and in 63% samples enriched with soil extract and PSM (Table 3). These observations are consistent with those of Bælum et al. (2006), who found that bacteria harboring *tfdA Class III* genes were abundant during degradation of MCPA. *Class I* gene amplification products, in turn, were only observed in 19% of samples, despite the use of modified PCR conditions (Table 3).

**Table 3:** PCR results for target genes; “ +” presence of PCR product on 1.5% agar gel electrophoresis.

**Days of incubation**	**MCPA concentration**	**Target gene**											
		**With soil extract**						**With soil extract and PSM (syringic acid)**					
		***16S rRNA***	***tfdA***	***tfdA alfa***	***tfdA cI***	***tfdA cII***	***tfdA cIII***	***16S rRNA***	***tfdA***	***tfdA alfa***	***tfdA cI***	***tfdA cII***	***tfdA cIII***
6	0.1 mM	+						+		+		+	+
12			+						+					+
18			+						+					+
24		+						+				+	
6	0.5 mM	+		+		+	+	+					
12			+					+	+		+	+	+	
18			+					+	+		+	+	+	+
24		+		+	+	+		+		+		+	+

**Notes.**

PSM Plant Secondary Metabolite

The obtained variety of *tfdA*-like genes suggests that a highly diverse mix of bacteria capable of degrading MCPA were present in the samples. The bacteria harboring *tfdA*-like genes can be divided into three groups based on their phylogeny and catabolic gene diversity. The first group, consisting of copiotrophic β- and γ-proteobacteria (Zhou, Wu & Xiang, 2014), carry *tfdA* genes encoding α-ketoglutarate-dependent dioxygenase, which is involved in the first step of the phenoxy herbicide degradation pathway (Batogliu-Pazarbasi et al., 2013). This group is subdivided into three classes based on the sequences of the gene: *Class I* sequences, found in the *Burkholderia*-like, *Comamonas-Rhodoferax* and *Cupriavidus-Alcaligenes* related organisms of the β-proteobacteria; *Class II* sequences are less widely distributed, being found only in *Burkholderia*-like branches; *Class III* sequences found in the *Comamonas-Rhodoferax* group of γ-proteobacteria. The second and third groups include α-proteobacteria harboring the *tfdAα* gene (Itoh et al., 2004), these being closely related to *Bradyrhizobium* spp. in the second group, and to *Sphingomonas* in the third one.

**Figure 1:**
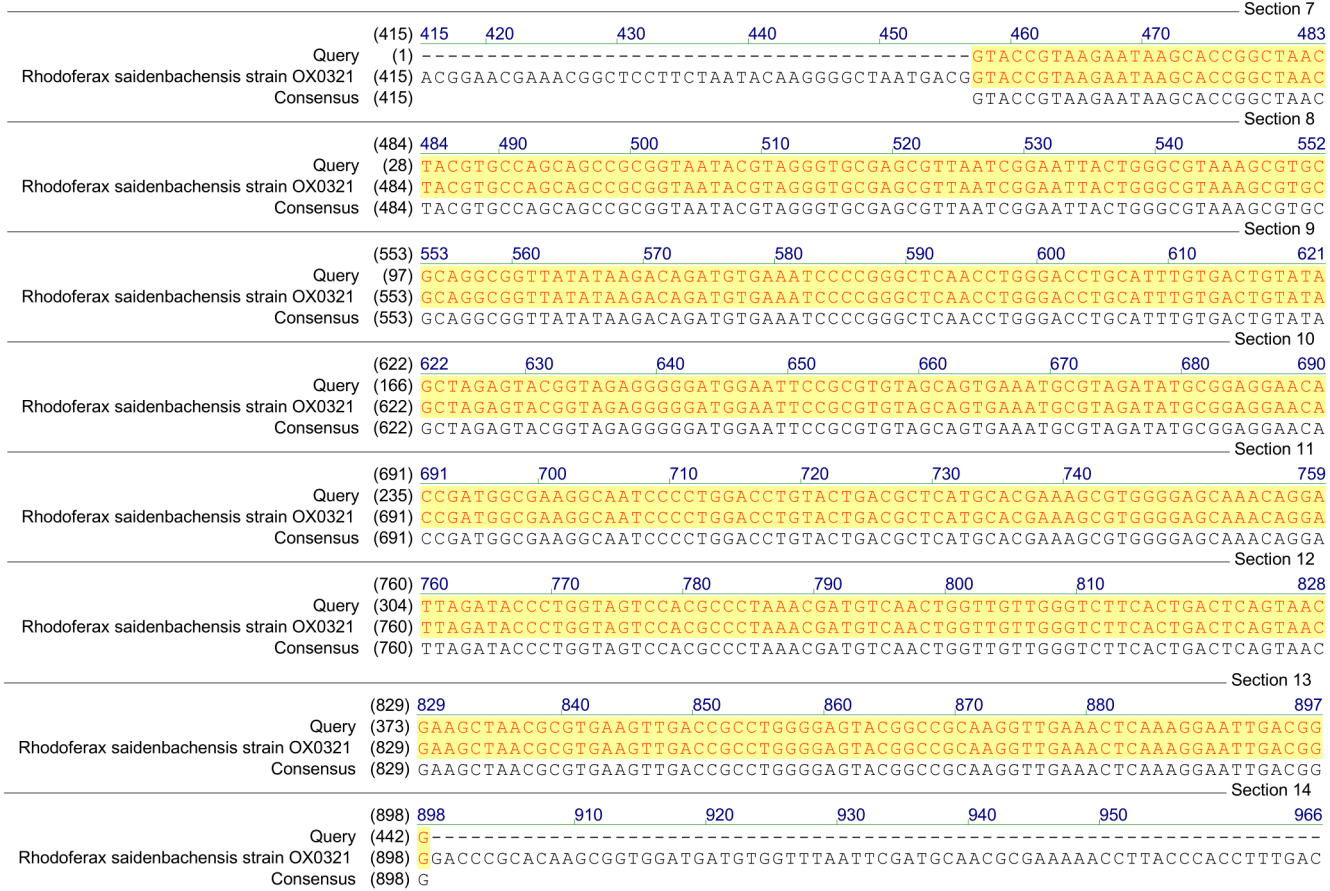
Homology analysis of 16S rRNA gene fragment (1,300–1,400 bp) amplified in samples enriched with MCPA and syringic acid (Query—obtained sequence).

**Figure 2:**
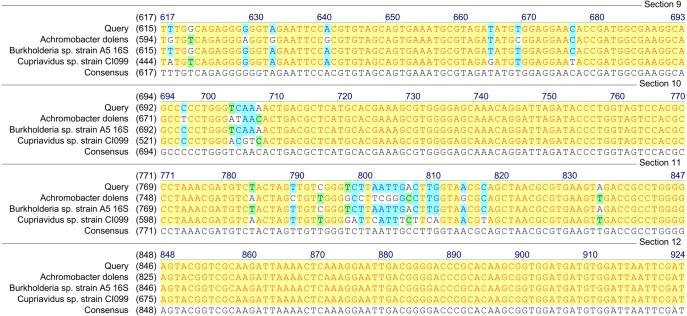
Homology analysis of 16S rRNA gene fragment (1,300–1,400 bp) amplified in samples enriched with MCPA and syringic acid (Query—obtained sequence).

Based on the results of *tfdA* genes presence, our findings indicate the occurrence of almost all three groups of bacteria in the studied samples. However, in order to confirm the biodiversity, the 16S rRNA sequences analyses have been performed. The obtained results revealed ubiquitous enrichment of several sequence variants affiliated with microbial taxa which have been reported to act as degraders of many classes of environmental pollutants. These include members of the genus *Rhodoferax, Achromobacter, Burkholderia* and *Cupriavidus* previously found to be involved in the degradation of phenolic and chloroaromatic compounds, including phenoxy herbicides (Streber, Timmis & Zenk, 1987; Tonso, Matheson & Holben, 1995; Ehrig, Müller & Babel, 1997; Bælum, Jacobsen & Holben, 2010; Xia et al., 2017). All genera belong to the β-proteobacteria and, are considered to harbor *tfdA* genes. The highest, 100% (*E*-value = 0), homology was established with regard to the 16S rRNA gene of the *Rhodoferax saidenbachensis* strain OX0321 (GenBank accession no. MG576020.1) (Fig. 1), while homologies with the 16S rRNA genes of the *Achromobacter dolens* strain BFHC1 5 (GenBank accession no. MG897148.1), *Burkholderia* sp. strain A5 (GenBank accession no. KY623377.1) and *Cupriavidus* sp. strain CI099 (GenBank accession no. MG798754.1) were found to be 99% with *E*-values being respectively 1e−44, 1e−44 and 4e−44 (Fig. 2). Obtained results are consistent with data from other studies of MCPA-degrading strains belonging to β-proteobacteria including inter alia *Cupriavidus necator* JMP134 (Streber, Timmis & Zenk, 1987), *Cupriavidus* sp. (Tonso, Matheson & Holben, 1995), *Burkholderia* sp. (Bælum, Jacobsen & Holben, 2010), *Rhodoferax* sp. (Ehrig, Müller & Babel, 1997), *Achromobacter* sp. LZ35 (Xia et al., 2017), and *Achromobacter xylosoxidans* (Tonso, Matheson & Holben, 1995).

The results also show that simultaneous application of herbicide and PSM contributes to increased detection of MCPA degradative genes. Samples spiked with both MCPA and syringic acid demonstrated a mean two-fold increase of detected genes in comparison to those amended only with MCPA. In the case of *tfdAα*, the number of genes was found to double when PSM was applied; the same was true for the *tfdA Class I* gene. Detection of *tfdA Class II* demonstrated the greatest (2.5-fold) increase, while *tfdA Class III* showed the smallest (1.7-fold) increase when PSM was added (Table 3). Such an increase associated with *tfdA*-like genes demonstrates that syringic acid can enhance the biodegradation of structurally-related MCPA.

Moreover, it was found that samples spiked with a lower dose of MCPA (0.1 mM) demonstrated a lack of any *tfdA*-like genes, while in those spiked with syringic acid, three of five genes (*tfdAα*, *tfdA Class II* and *tfdA Class III*) were amplified, being detected in 30% of samples (Table 3). Greater detection was observed in the case of samples treated with 0.5 mM MCPA: the genes were detected in 45% of all samples, i.e., 40% when PSM was not added and 50% when it was supplemented (Table 3). In addition, samples with higher concentration of MCPA and no syringic acid, showed the occurrence of *tfdA* genes at 6th and then 24th day of experiment; while in samples spiked with syringic acid functional *tfdA* genes were amplified starting from 12th day of incubation. This can be related to the anti-microbial effect of syringic acid (Cheemanapalli et al., 2018), that may influence on depletion of genes occurrence in the initial phase of experiment.

However, not only bacteria have been found to degrade phenoxy acid herbicides: similar observations have been made in fungi (Poll et al., 2010). In a study of 90 fungal strains, Vroumsia et al. (2005) found 52% of spiked phenoxy herbicides to be degraded by single fungal strains in five days. In contrast to bacteria, no distinct pathway of fungal phenoxy herbicide degradation is known. It is suggested that the degradation process may be performed by non-specific enzymes like peroxidases and laccases (Del Pilar Castillo et al., 2001). As whole soil extract including soil bacteria and fungi was used for the present study, it is possible that beside bacteria whose presence was confirmed by the presence of 16S rRNA and functional degradative *tfdA* genes (Table 3), the observed MCPA depletion may also be attributed to the action of fungi.

### The influence of syringic acid on the efficiency of MCPA removal

Numerous kinetic models have been developed to describe the mineralization of xenobiotics in the environment (Itoh et al., 2004; Bælum et al., 2006; Nicolaisen et al., 2008). These are based on diverse forms of degradation kinetics which may, or may not, take into account microorganism activities. Moreover, various other models of microorganism growth have been developed depending on initial cell biomass density and substrate concentration (Nielsen et al., 2011). Most studies examining the efficiency of herbicide removal describe the role of single strains or known bacterial consortia intentionally transferred to the test medium (Itoh et al., 2004; Bælum et al., 2006; Nicolaisen et al., 2008; Müller et al., 1999).

**Figure 3:**
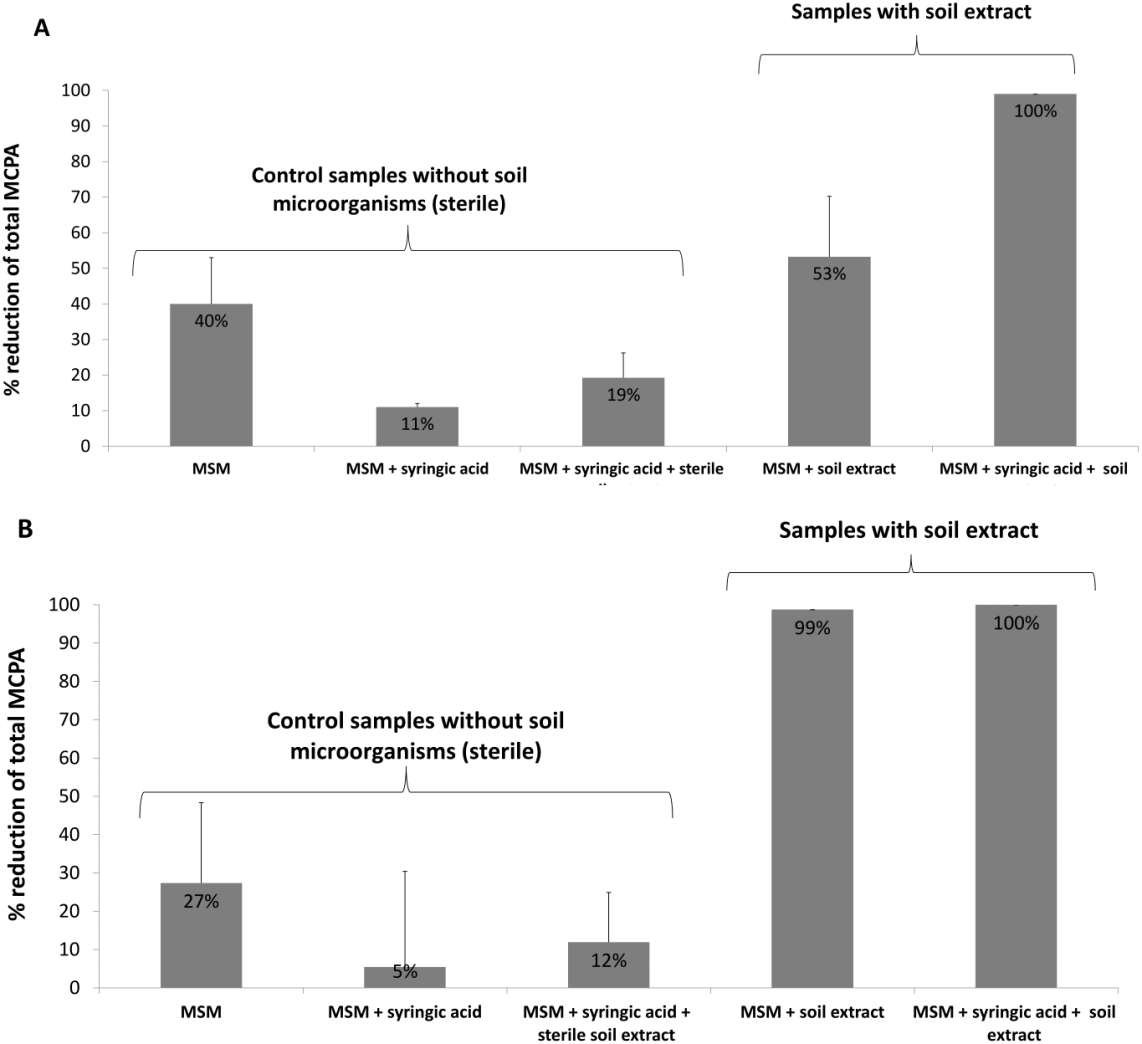
Percentage reduction of total MCPA concentration during the 24-day incubation period: (A) MCPA spiked in concentration of 0.1 mM; (B) MCPA spiked in concentration of 0.5 mM.

In the case of the present study, microbial biomass was introduced to the system by a soil extract containing bacteria, fungi and other microorganisms. In addition to the soil microflora, some samples were also enriched with a PSM (syringic acid) to increase the efficiency of MCPA removal. The results indicate a 40% reduction in herbicide content in MSM samples amended with 0.1 mM MCPA after 24 days of incubation; however, the addition of soil microorganisms with the soil extract enhanced the removal rate to 53%, and the simultaneous application of both soil extract and syringic acid boosted the process to 100% (Fig. 3A). Samples amended with 0.5 mM MCPA demonstrated 27% MCPA removal when only MSM was used and 99% when soil extract was applied. Combined soil extract and syringic acid application increased MCPA removal to 100% (Fig. 3). Similar results were observed by Fraraccio et al. (2017), who noted increased degradation of cis-1,2-dichloroethylene in the presence of selected PSMs. Several studies have also demonstrated that PSMs play a positive role in PCB and PAH biodegradation (Singer, Thompson & Bailey, 2004; Jha, Panwar & Jha, 2015).

The observed reductions of initial MCPA concentration in the control samples can be related to both: abiotic degradation of the herbicide as well as it sorption on the salts (containing Fe and Ca) used for MSM preparation (Kersten et al., 2014). Depletion of MCPA in samples spiked with syringic acid and sterile soil extract can be the result of sorption of MCPA on humic acids introduced with soil extract (Ćwieląg-Piasecka et al., 2018).

It is also worth to underline that the samples amended with a higher concentration of MCPA demonstrated greater herbicide removal regardless of whether they contained or did not contain microorganisms; this is also consistent with the results of the molecular analysis, which revealed the presence of a higher number of degradative *tdfA*-like genes when MCPA was used at the higher concentration (0.5 mM) (Table 3). The addition of PSM further increased the number of detected genes (Table 3), and at the same time increased the removal of MCPA when lower herbicide concentration was used (Fig. 3A). Application of higher MCPA concentration together with PSM, in turn, did not significantly change the herbicide removal rate (Fig. 3B).

### Influence of MCPA and syringic acid on sample phytotoxicity

A key consideration in the performance of toxicological analysis is the fact the true risk associated with environmental contaminants cannot be determined simply by conventional chemical analysis. This can be attributed to the range of additive, synergistic, antagonistic and potentiating interactions that occur between the environmental compounds which influence the final status of a given sample. Toxicity tests (biotests) such as Phytotoxkit allow an effective assessment of the toxicity of the tested sample.

Although MCPA has been used since 1940s, almost no attention has been paid to its toxicological effects on terrestrial ecosystems. The acute toxicity of this pollutant has been determined on the laboratory scale using individual bacteria, crustaceans, green algae and mammalian cells. These findings concern genotoxicity and carcinogenicity, but not seed germination or shoot and root growth inhibition (Pereira, Cerejeira & Espírito-Santo, 2000; Grabińska-Sota, Wiśniowska & Kalka, 2003).

Considering the above, the present work was undertaken to study the toxicological effect of MCPA on vascular plants, measured at the time of herbicide application and after a 24-day incubation. The species tested were the broad-leaved plants *L. sativum* and *S. alba*. It was found that the ongoing physicochemical and microbial degradation processes significantly reduced the phytotoxicity of the samples, falling from 100% inhibition of root length for all samples and both plant species at the beginning, to high stimulation of root growth after 24 days (range from −21% to −102%) (Table 4). Mierzejewska, Baran & Urbaniak (2018) also noted high levels of phytotoxicity in soil freshly amended with MCPA, i.e., 99% inhibition for *L. sativum* and 97% for *S. alba*. However, root length inhibition dropped to 3% for *L. sativum* and 34% for *S. alba* after a three-week incubation period, presumably due to the combined action of physicochemical transformation processes and biodegradation by indigenous soil bacteria (Mierzejewska, Baran & Urbaniak, 2018). In both, the present study and that of Mierzejewska, Baran & Urbaniak (2018), it was found that, despite both test plants demonstrating similar initial high phytotoxic responses, *S. alba* was more sensitive to the MCPA residues remaining after the incubation period (Table 4). Similarly, Urbaniak et al. (2016) and Grabińska-Sota, Wiśniowska & Kalka (2003) reported *S. alba* to have greater sensitivity to MCPA than *L. sativum*.

**Table 4:** Changes in sample phytotoxicity after 24 days of incubation.

***Variant***		***Inhibition (%)***			
		***Beginnning***		***After 24 days***	
		***L.sativum***	***S.alba***	***L.sativum***	***S.alba***
Sterile samples, without microorganisms	MSM + 0.1mM MCPA	100	100	100	100
	MSM + 0.5mM MCPA	100	100	100	100
	MSM + 0.1mM MCPA + PSM (SA)	100	100	100	100
	MSM + 0.5mM MCPA + PSM (SA)	100	100	100	100
	MSM + 0.1 mM MCPA + PSM (SA) + Soil Extract	100	100	100	100
	MSM + 0.5 mM MCPA + PSM (SA) + Soil Extract	100	100	100	100
Not-sterilized samples, with microorganisms originated from soil extract	MSM + 0.1 mM MCPA + Soil Extract	100	100	−102^a^	−34^a^
	MSM + 0.5 mM MCPA + Soil Extract	100	100	−47^a^	−38^a^
	MSM + 0.1 mM MCPA + PSM (SA) + Soil Extract	100	100	−69^a^	−21^a^
	MSM + 0.5 mM MCPA + PSM (SA) + Soil Extract	100	100	−55^a^	−35^a^

**Notes.**

a Negative values indicate the stimulation of the test plants root growth in reference to control sample.

MSM Mineral Salt Medium PSM Plant Secondary Metabolite SA syringic acid

MCPA has been found to have negative effects on other plant species. For example, Podolska (2014), showed that MCPA increased soil phytotoxicity to buckwheat (*Fagopyrum esculentum* cv. Kora), causing stem deformation and discoloration of leaves, while Polit et al. (2014) demonstrated that MCPA had a toxic effect on seed germination and seedling development of winter oilseed rape (*Brassica napus*).

Our findings indicate that PSM application has a positive influence on the mitigation of the phytotoxicity of the samples; however, this was only observed when a higher dose of MCPA was used. In this case, the application of syringic acid led to higher stimulation of root growth (−55%) than in samples without PSM (−47%). The opposite effect was observed for the lower dose of MCPA: in this case, PSM application stimulated root growth to a lower extent: −102% vs. −69% for *L. sativum* and −34% vs. −21% for *S. alba* (Table 4). This can be related to the phytotoxic activity exhibited by syringic acid (Zhou, Wu & Xiang, 2014).

## Conclusions

To conclude, the obtained molecular, chemical and phytotoxicity assessment results demonstrate that the application of PSMs can positively influences the removal of structurally-related herbicides. The results confirm that spiking with syringic acid is associated not only with enhanced MCPA removal and hence decreased phytotoxicity, but also the detection of a higher number of genes responsible for MCPA biodegradation.

##  Supplemental Information

10.7717/peerj.6745/supp-1 File S1 Concentrations and reductions of MCPA The file contains the raw data of MCPA cocnetrations.

10.7717/peerj.6745/supp-2 File S2 Roots length calculations for *l. sativum* and *S. alba* The file contains the raw data of roots lengh of *L. sativum* and *S.alba*.

## Additional Information and Declarations

### Data Availability

The following information was supplied regarding data availability: The raw data is available in Files S1 and S2.
